# Optimizing density of intrathecal contrast at digital subtraction myelography: Evaluation of differing needle characteristics and injection rates in a phantom

**DOI:** 10.1177/15910199251375541

**Published:** 2025-09-16

**Authors:** Anahita Malvea, Emily Chung, Mehran Nasralla, Eef J. Hendriks, Richard I. Farb

**Affiliations:** 1 Department of Medical Imaging, 7938 University of Toronto, Canada; 2 Department of Medical Imaging, Division of Neuroradiology, 26625 University of Toronto, Canada

**Keywords:** Digital subtraction myelography, intracranial hypotension, cerebrospinal fluid-venous fistulas, spine procedures

## Abstract

Dynamic myelography, performed as digital subtraction myelography or dynamic computed tomography myelography, is crucial in diagnosing intracranial hypotension resulting from a cerebrospinal fluid-venous fistula (CVF). The quality of the myelogram is paramount for accurate diagnosis. Using a phantom, the impact of needle type (Quincke vs. Whitacre), caliber, side-hole position, and rate of injection on the quality of the myelogram was determined. The ideal decubitus myelogram would provide a large volume of a hyperdense contrast within the lateral dependent aspect of the thecal sac, optimally flooding the mouths of the neural foramina and root sleeves where the vast majority of CVFs originate. The results of this study suggest it is exclusively the rate of injection that most predictably dictates the quality of the myelogram in this regard. Specifically, a slow injection rate, on the order of 0.1 mL/s, should be opted for to decrease turbulence, optimize myelogram quality, and thus improve CVF detection in clinical practice.

## Introduction

Digital subtraction myelography (DSM) has been essential in both diagnosing and guiding management of intracranial hypotension resulting from cerebrospinal fluid (CSF)-venous fistula (CVF).^1–3^ However, the literature suggests that in the process of performing a DSM with a Quincke needle, iatrogenic CSF leaks may occur.^4–6^ Anecdotal experience with the Quincke and the Whitacre needles of various gauges suggests variability in the quality of the DSM, which can lead to prolonged procedures and associated risks. The ideal decubitus myelogram would provide a large volume of a hyperdense layer of contrast within the lateral, dependent aspect of the thecal sac (“gutter”), thereby optimally covering and flooding the neural foramina and nerve root sleeves, where the vast majority of CVFs originate. By increasing the volume, uniformity, and density of this hyperdense layer of contrast, it is anticipated that the conspicuity of a CVF will rise, increasing sensitivity to detection. This has been evidenced in computed tomography myelography by Edelmuth et al.,^7^ who demonstrate that intrathecal contrast attenuation is positively associated with CVF visibility in their study of 24 patients. Therefore, this study is aimed at identifying variables that maximize the hyperdense layer of contrast agent.

## Methods

**Figure 1.**
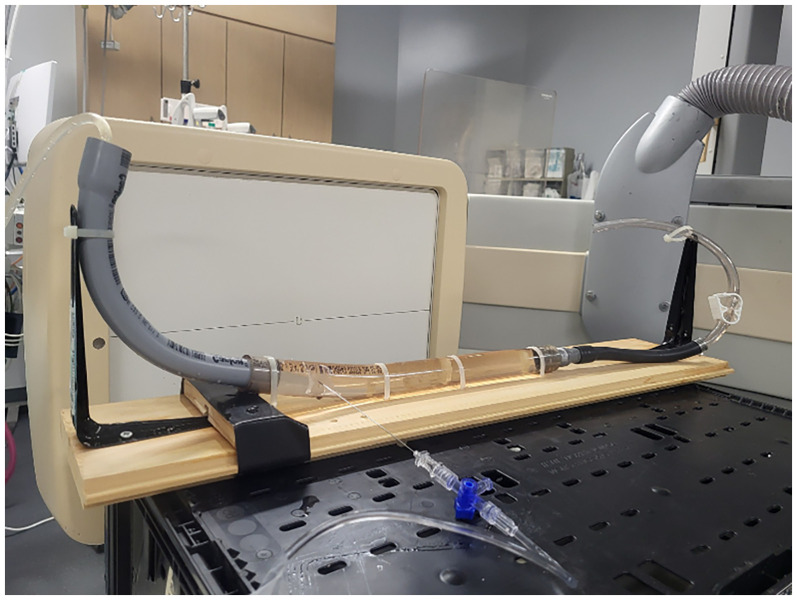
Experimental setup of the polyethylene phantom with lateral biplane imaging on the Phillips Allura Neuroangiography table.

**Figure 2.**
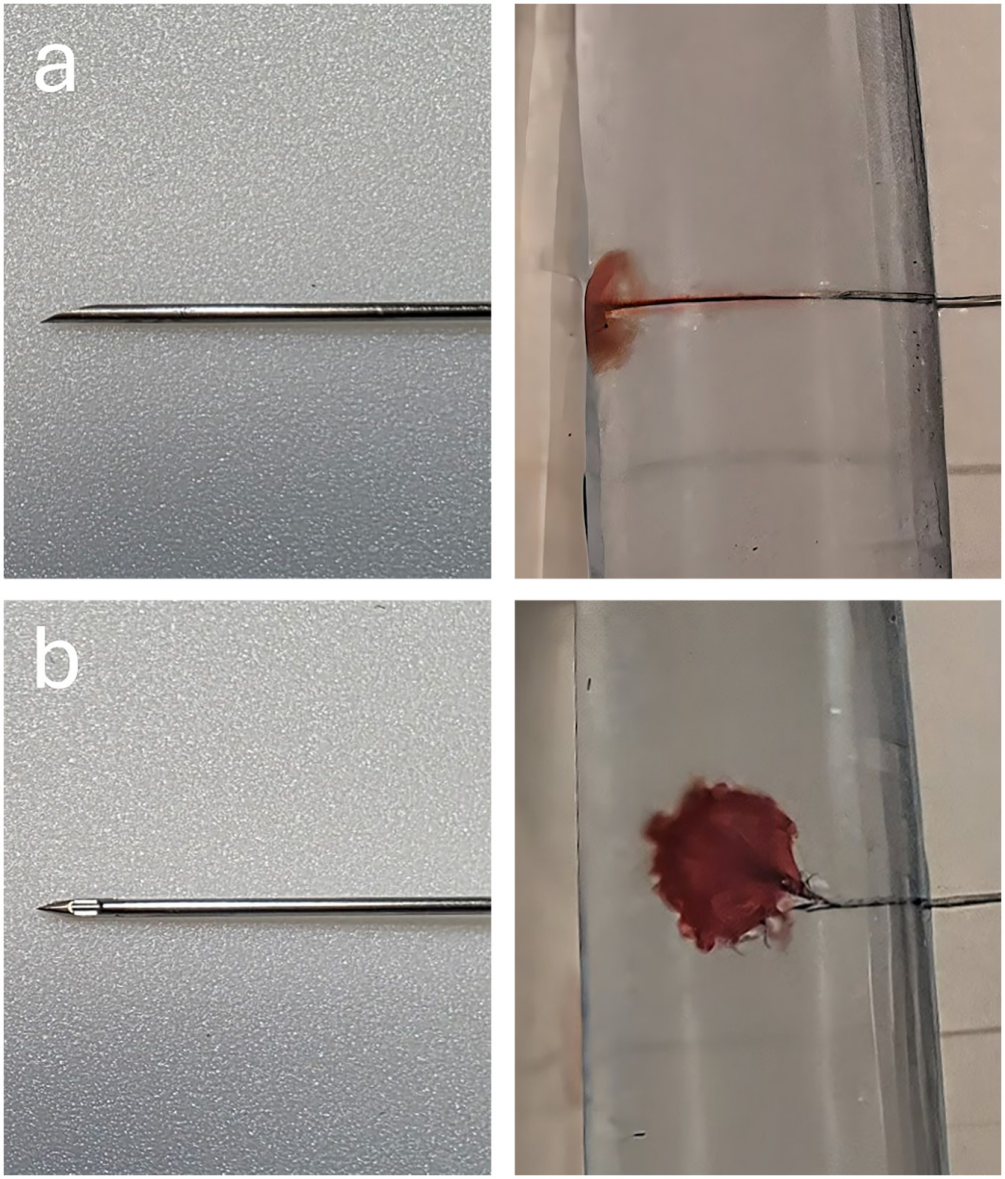
The (a) Quincke needle has a beveled cutting edge and the (b) Whitacre needle has a blunt tip with a side-hole originating from the lateral wall of the needle. The non-directional and directional injections are shown, respectively.

A phantom (Figure 1) was created to replicate the thecal sac. This was fabricated using polyethylene tubing with an internal diameter of 2 cm. Between experimental angiographic runs, the phantom was filled and cleansed repeatedly with saline and refilled to ensure no residual contrast was present to confound subsequent runs. Both 22- and 25-gauge Quincke (Becton Dickson) and Whitacre (Becton Dickson) needles were used (Figure 2). A Medrad injector (Bayer HealthCare, Whippany, New Jersey) was used for each run with a rate rise of 1 s. A total of 5 mL of Iohexol (Omnipaque 300; GE Healthcare, Piscataway, New Jersey) was used for each injection. All needles were tested with injection rates of 0.1, 0.2, 0.3, 0.4, 0.5, and 1.0 mL/s. Imaging was performed using a Philips Allura angiography system in a single, lateral plane at a rate of 1 frame per second for up to 45 s (dictated by visual determination of equilibrium).

The Quincke needle has a unidirectional exit point, while the Whitacre needles (a version of a side-hole needle) were tested with the side-hole pointed to the 12, 3, 6, and 9 o’clock positions (12 being aimed superiorly and 9 being cranially oriented).^8,9^ Qualitative assessment of the initial injection pattern/directionality, the appearance of the contrast column, as well as quantitative measurements, in the form of pixel region of interest (ROI) and contrast column thickness, were performed to determine the impact of the tested variables on the quality of the myelogram. The qualitative assessment of the injection was conducted by two members of the team independently. Quantitative measurements were performed independently by a single member of the team.

## Results

Qualitatively, the Quincke needle injection stream was seen to be unidirectional (Figure 2). The Whitacre needle injects in the direction the side-hole is pointed, at high injection rates, the needle pointed to 3 o’clock and 9 o’clock demonstrates a parabola-shaped injection stream, at slower injection rates, contrast flows at a 45° angle from the side-hole.^8,9^

**Figure 3.**
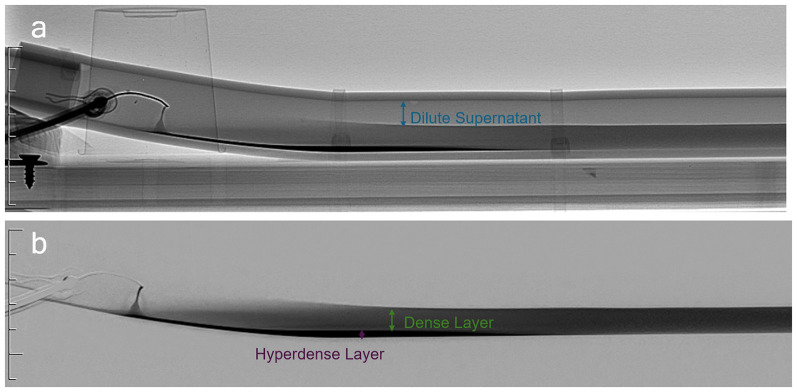
(a) Fluoroscopic image of the phantom and (b) digitally subtracted image of the contrast column, demonstrating the stratified three distinct layers of: dilute supernatant, dense layer, and hyperdense layer (desired).

All injection rates of more than 0.2 mL/s resulted in a three-layer contrast column consisting of a dilute supernatant, dense, and hyperdense layer (layer of interest in optimizing the myelogram) (Figure 3). This typical 3-layered dispersion occurred regardless of needle size, type, and orientation.

**Figure 4.**
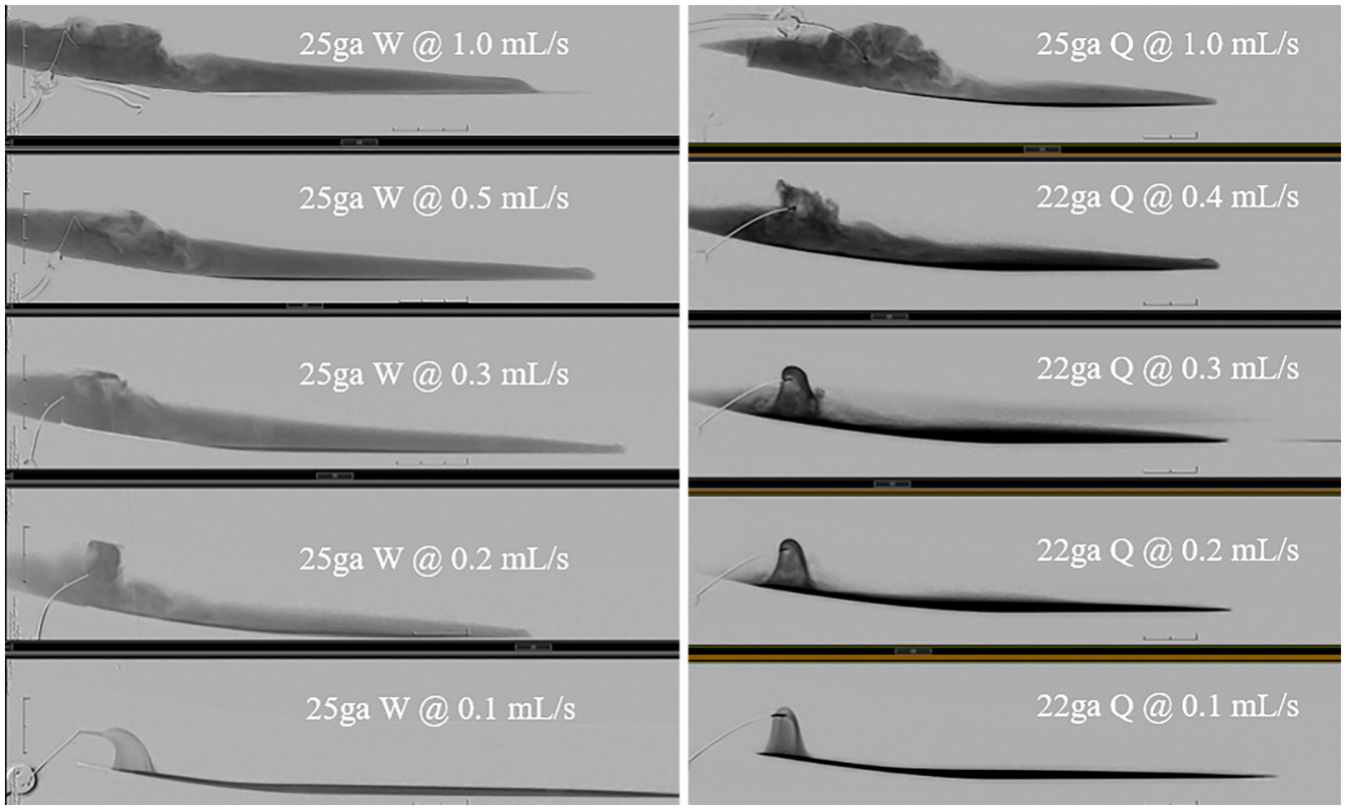
Assorted examples of mid-injection images of various combinations of needle type, gauge, and injection rates. Note the lowest injection rates of 0.1 mL/s resulting in the least “burst-like”/turbulent mixing of saline and contrast. This demonstrates the most concentrated hyperdense layer without contrast dilution.

Comparing the average thickness of the dense layer of contrast at injection rates of 0.5 and 1.0 mL/s for needles of varying calibers, the Quincke and Whitacre needles, and varied direction of the side-hole, a difference of <1 mm was seen—observably negligible. A minimal difference was also observed with the average thickness of the hyperdense layer. Similarly, comparing the average pixel value across experiments yielded a difference of <100 pixels (Table 1). Overall, no visually observable difference was seen in the thickness or pixel ROI with varying needle gauge, needle type, or direction of the side-hole (Whitacre needle) at flow rates of 0.2 mL/s or more (Figure 4).

**Table 1.** Average thickness of the dense and hyperdense layers; average pixel value of the dense and hyperdense layers across experimental runs.

	Average thickness of dense layer (mm)	Average pixel value^*^ of dense layer	Average thickness of hyperdense layer (mm)	Average pixel value^*^ hyperdense layer
Averages of experiments performed at 0.5 and 1.0 mL/s				
Quincke 25	0.93	355.00	0.27	8.90
Quincke 22	0.92	267.50	0.24	3.55
Whitacre 25 @ 12	1.03	335.50	0.23	25.20
Whitacre 25 @ 3	1.05	309.00	0.28	12.45
Whitacre 25 @ 6	0.79	279.50	0.25	17.35
Whitacre 25 @ 9	1.01	303.25	0.21	10.15
Whitacre 22 @ 12	0.92	278.50	0.30	9.45
Whitacre 22 @ 3	0.94	332.50	0.28	16.50
Whitacre 22 @ 6	0.68	244.50	0.31	2.10
Whitacre 22 @ 9	0.94	249.50	0.29	3.35
Average results at varied flow rates				
**0.1 mL/s**	**0**. **10**	**654**. **43**	**0**. **44**	**0**. **23**
**0.2 mL/s**	**0**. **58**	**553**. **88**	**0**. **43**	**2**. **94**
0.3 mL/s	0.95	385.75	0.39	1.94
0.4 mL/s	0.99	283.13	0.32	12.01

*Pixel value is the inverse of contrast density; higher pixel values represent brighter regions (less contrast).

A qualitative assessment revealed that at injection rates of >0.2 mL/s there was a “burst” (Figure 4) of contrast, which led to turbulence and mixing of saline and contrast, therefore, resulting in the typically encountered 3-layered contrast column. These qualitative findings were visually obvious with significant differences between a “burst” injection and a “non-burst” injection; thus, there was little risk of differences in interpretation between observers. This burst was maximal at higher injection rates of 1.0 mL/s but nonetheless occurred at all injections employing a rate >0.2 mL/s.

In addition to the assessment of needle caliber, type, and side-hole direction, experiments were conducted to isolate the impact of injection rate. Using the 22-gauge Quincke and Whitacre needle, pointed to 9 o’clock, injection rates of 0.1–0.4 mL/s were tested. Using the 25-gauge Quincke and Whitacre needles, as well as a single 27-gauge Whitacre needle, a rate of injection of 0.1 mL/s was tested.

Flow rates below 0.1 mL/s were not tested, given that 0.1 mL/s is the lower limit of the Medrad injector. In addition to this technical limitation, injection rates of <0.1 mL/s will increase the injection time beyond 100 s, which may not be desirable for clinical myelography.

**Figure 5.**
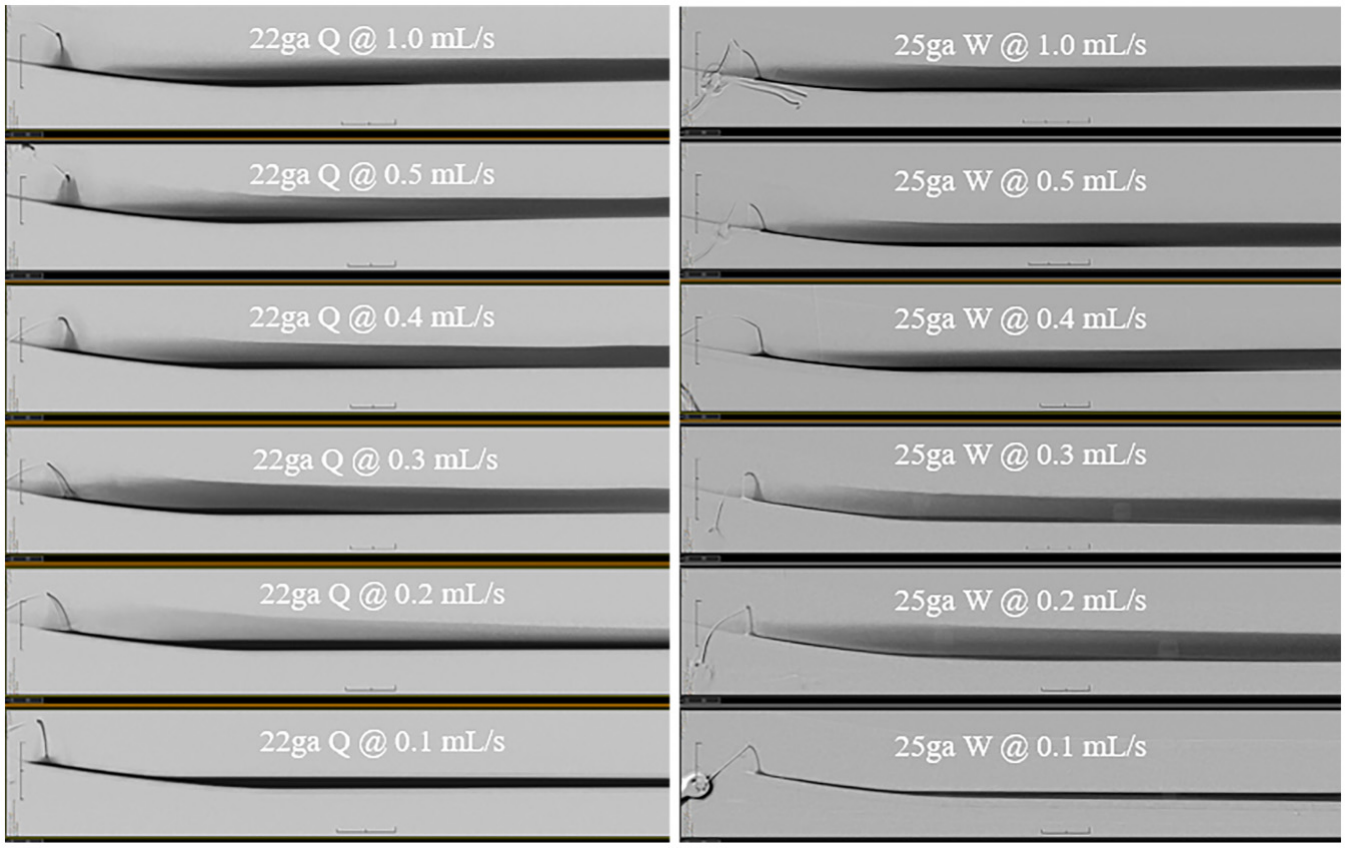
Assorted examples of end-injection images of various combinations of needle type, gauge, and injection rates. Note the commonly seen three distinct layers of contrast stratification, that is, supernatant saline, dense layer, and hyperdense layer. The lowest injection rate of 0.1 mL/s results in an optimized single layer of hyperdense contrast regardless of needle type.

A qualitative assessment demonstrated noticeably varied results between injection rates. No turbulence of contrast was seen at 0.1  and 0.2 mL/s. There was a clear elimination of the middle dense layer or, at the very least, a decrease in density/height of the middle dense layer at 0.1 mL/s, with a faint layer at 0.2 mL/s (Figure 5). Qualitative results were reinforced by quantitative measurements of the ROI and thickness of the contrast layers (Table 1): a difference in dense layer thickness of >4 mm between varied injection rates was seen. An evident difference was also seen with average pixel values varying by >300 pixels. These experiments suggest that the lowest achievable injection rate, in this case 0.1 mL/s, demonstrates the ideal contrast deposition. The finding of a single hyperdense column of contrast was replicated with all needle calibers (22-, 25-, and 27-gauge) and types (Quincke and Whitacre), which were tested, without an appreciable difference in the myelogram with a 0.1 mL/s injection rate. The 27-gauge needle, which is the least traumatic, demonstrated the desired myelogram at 0.1 mL/s. However, other injection rates were not attempted, given the small caliber of the needle.

The limitations in extending the results of this study to clinical practice include the limits of a phantom model in replicating CSF-contrast dynamics seen in vivo. Therefore, in vivo assessment of the suggested method is required.

## Conclusions

These experiments reveal that it is the rate of injection of contrast which is the key variable responsible for improving the quality of the myelogram with regard to increasing the density and uniformity of the contrast agent layering in the dependent portion of the thecal sac. The needle type, size, and direction of injection (for Whitacre needles) have herein been shown to be inconsequential in this regard.

Injection rates on the order of 0.1 mL/s are recommended when performing myelography for the detection/localization of CVF. This low injection rate will allow contrast to be deposited in the lateral, dependent margin of the thecal sac with limited turbulence and mixing and consequently decreased dilution. The higher density of the contrast agent in the ROI is anticipated to result in increased conspicuity of CVFs and overall greater accuracy of the exam (7). Moreover, since a slow injection is mandated, the use of smaller gauge spinal needles is now encouraged. This also allows for 25- and 27-gauge side-hole needles to be used to minimize the risk of post-dural puncture headaches associated with dynamic myelography.

Prior to the results of these phantom experiments, a combination of 22-gauge Quincke and Whitacre needles was being used, with increasing preference for the Whitacre needles given recent evidence of decreased iatrogenic CSF leak and post-dural puncture headaches. Fast injections were implemented in order to achieve optimal subtraction on DSM; high injection rates previously limited the use of smaller gauge needles. However, with mounting evidence of increased contrast density providing optimal myelograms, the need for good subtraction images is less of a necessity.^5^ These results allow for smaller, and therefore atraumatic, needle gauges to be used, given that the sole variable is the injection rate.

An initial implementation of the above technique has thus far yielded anecdotal, favorable clinical results for the detection of a CVF, thereby changing our clinical practice due to a substantial modification of the injection parameters listed in the literature to date.^10^ A subsequent rigorous analysis of the impact of slow injection myelography is anticipated.
